# Evaluation of the HOXA11 level in patients with lung squamous cancer and insights into potential molecular pathways via bioinformatics analysis

**DOI:** 10.1186/s12957-018-1375-9

**Published:** 2018-06-18

**Authors:** Rui Zhang, Tong-tong Zhang, Gao-qiang Zhai, Xian-yu Guo, Yuan Qin, Ting-qing Gan, Yu Zhang, Gang Chen, Wei-jia Mo, Zhen-bo Feng

**Affiliations:** 1 Department of Pathology, First Affiliated Hospital of Guangxi Medical University, 6 Shuangyong Road, Nanning, 530021 Guangxi Zhuang Autonomous Region People’s Republic of China; 2 Department of Medical Oncology, First Affiliated Hospital of Guangxi Medical University, 6 Shuangyong Road, Nanning, 530021 Guangxi Zhuang Autonomous Region People’s Republic of China

**Keywords:** HOXA11, Lung squamous cancer, qRT-PCR, Clinical features, Enrichment analysis

## Abstract

**Background:**

This study was carried out to discover the underlying role that HOXA11 plays in lung squamous cancer (LUSC) and uncover the potential corresponding molecular mechanisms and functions of HOXA11-related genes.

**Methods:**

Twenty-three clinical paired LUSC and non-LUSC samples were utilized to examine the level of HOXA11 using quantitative real-time polymerase chain reaction (qRT-PCR). The clinical significance of HOXA11 was systematically analyzed based on 475 LUSC and 18 non-cancerous adjacent tissues from The Cancer Genome Atlas (TCGA) database. A total of 102 LUSC tissues and 121 non-cancerous tissues were available from Oncomine to explore the expressing profiles of HOXA11 in LUSC. A meta-analysis was carried out to further assess the differential expression of HOXA11 in LUSC, including in-house qRT-PCR data, expressing data extracted from TCGA and Oncomine databases. Moreover, the enrichment analysis and potential pathway annotations of HOXA11 in LUSC were accomplished via Gene Oncology (GO) and Kyoto Encyclopedia of Genes and Genomes (KEGG). The expression of hub genes and according correlations with HOXA11 were assessed to further explore the biological role of HOXA11 in LUSC.

**Results:**

HOXA11 expression in LUSC had a tendency to be upregulated in comparison to adjacent non-cancerous tissues by qRT-PCR. TCGA data displayed that HOXA11 was remarkably over-expressed in LUSC compared with that in non-LUSC samples, and the area under curves (AUC) was 0.955 (*P* < 0.001). A total of 1523 co-expressed genes were sifted for further analysis. The most significant term enriched in the KEGG pathway was focal adhesion. Among the six hub genes of HOXA11, including PARVA, ILK, COL4A1, COL4A2, ITGB1, and ITGA5, five (with the exception of COL4A1) were significantly decreased compared with the normal lung tissues. Moreover, the expression of ILK was negatively related to HOXA11 (*r* = − 0.141, *P* = 0.002).

**Conclusion:**

High HOXA11 expression may lead to carcinogenesis and the development of LUSC. Furthermore, co-expressed genes might affect the prognosis of LUSC.

## Background

Non-small cell lung cancer (NSCLC) has caused the most frequently cancer-related deaths among all types of malignancy in humans worldwide, accompanied by a high incidence [1–3]. NSCLC is responsible for the majority of the primary lung cancer cases, including large cell carcinoma, lung adenocarcinoma (LUAD), and lung squamous cancer (LUSC). Among the three histological subtypes, LUSC is the most common type in developing countries [4–7], and patients with lung cancer are still facing a low overall 5-year survival rate [8, 9]. Molecular targeted therapy has achieved curative efficacy in clinical in LUSC. For example, EGFR targeted therapy had a modest effect in advanced LUSC patients [10]. However, the number of applicable patients is limited [11]. Thus, there is an urgent need to identify more underlying high-performance targets in LUSC.

Cumulative evidence has demonstrated that HOX genes, which belong to the large family of homeodomain genes, work to regulate growth processes, such as organogenesis and body patterning [12, 13]. Humans have HOX genes in four clusters (HOXA, HOXB, HOXC, and HOXD) [14, 15]. Cumulative studies have reported that HOX genes are expressed in healthy human lungs and play a crucial role in their development [12]. The HOXA11 cluster is located on chromosome 7p15-7p14.2, and 12 genes are involved in the cluster, including EVX1 and 11 HOX genes [16, 17]. A number of studies have been carried out to define the function of HOXA genes in malignant cancers. An increasing number of reports suggest that HOXA11 has been implicated in several malignant tumors, such as gastric cancer [18], renal cell carcinoma [19], NSCLC and lung adenocarcinoma [17, 20], and breast cancer [21]. Thus far, the expression level of HOXA11 and its potential mechanisms in LUSC have not been clarified.

In the present study, we attempted to identify the association between clinical parameters and HOXA11 expression to gain a comprehensive understanding of the role of HOXA11 in LUSCs. The mechanisms of HOXA11 co-expressed genes were mined by bioinformatics analysis.

## Methods

### Selection of clinical LUSC tissue samples

Clinical samples were collected from 23 LUSC patients who had been pathologically identified at the Department of Pathology, First Affiliated Hospital of the Guangxi Medical University (Nanning, Guangxi, China), from January 2012 to February 2014. The clinicopathological features of the patients are shown in Table 1. The Ethical Committee of the First Affiliated Hospital of Guangxi Medical University approved the present research. All participating clinical doctors and patients signed written informed consents.

**Table 1:** Relationships between the expression of HOXA11 and clinicopathological parameters in LUSC

Clinicopathological parameters		*n*	Relevant expression of HOXA11 (2^-ΔCq^)		
			Mean ± SD	*t*	*P* value
Tissue	Adjacent non-cancerous lung tissue	23	0.347 ± 0.304	− 1.501^a^	0.138
	LUSC	23	0.764 ± 1.288		
Age (years)	< 60	15	0.892 ± 1.555	0.647^a^	0.525
	≥ 60	8	0.523 ± 0.529		
Gender	Male	18	0.850 ± 1.445	0.597^a^	0.557
	Female	5	0.455 ± 0.315		
Smoke	No	12	0.354 ± 0.255	− 1.655^a^	0.113
	Yes	11	1.211 ± 1.777		
Tumor size (cm)	≤ 3	7	1.401 ± 2.219	1.084^a^	0.319
	> 3	16	0.485 ± 0.438		
EGFR amplification	No	17	0.547 ± 0.477	− 0.829^a^	0.444
	Yes	6	1.379 ± 2.441		
EGFR protein	High	5	0.638 ± 0.613	0.242^a^	0.811
	Low	18	0.799 ± 1.433		
TNM	I–II	10	0.250 ± 0.188	− 1.756^a^	0.094
	III–IV	13	1.159 ± 1.621		
Vascular invasion	Yes	3	0.768 ± 0.535	− 0.006 ^a^	0.995
	No	20	0.763 ± 1.375		
Pathological grading	I	0		*F* = 0.574^b^	0.457
	II	16	0.900 ± 1.524		
	III	7	0.344 ± 0.130		

^a^Student’s paired or unpaired *t* test was used for comparison between two groups

^b^One-way analysis of variance (ANOVA) was performed

*P < 0.05 was considered statistically significant

### Total RNA isolation

Per the manufacturer’s instructions, we extracted total RNA with the miRNeasy FFPE Tissue Kit (QIAGEN, Shanghai, China). In addition, we detected the purity and concentration of total RNA using NanoDrop 2000 (ThermoScientific, USA).

### qRT-PCR assay

The 10 μl reaction system was set up so that the prepared total RNA could be reverse transcribed using a reverse transcription kit (ABI, Life Technologies, USA) based on the manufacturer’s instruction. Fluorochrome SYBR Green I Master was used for a 20-μl real-time fluorescence PCR system. The specific primers were as follows: HOXA11 forward primer: 5′-TGGTCCCTGCTCCTCTAAC-3′, reverse primer: 5′-GGCTCAATGGCGTACTCTC-3′ [22]. GAPDH (internal control) forward primer: 5′-TGCACCACCAACTGCTTA-3′, reverse primer: 5′-GGATGCAGGGATGATGTTC-3′. The expression difference was calculated using the 2^−△Cq^ method [23, 24].

### Data mining and analyzing

All clinicopathological parameters related to LUSC and mRNA (level 3) expression in LUSC were carefully downloaded from the TCGA Data Portal website (http://cancergenome.nih.gov). Based on the HOXA11 expression in LUSC, GraphPad Prism was applied to obtain the scatter diagram. In addition, SPSS was carried out to acquire receiver operating characteristic curves (ROCs) as well as overall survival (OS) and disease-free survival (DFS) curves. Meanwhile, the available data of HOXA11 expression in LUSC was mined in Oncomine (https://www.oncomine.org). Furthermore, we collected in-house qRT-PCR and data from public databases to gain insight into the differential expression of HOXA11 in LUSC using an integrative meta-analysis. The standard mean difference (SMD) was pooled from all studies to determine the expression level of HOXA11 in LUSC.

### Screening co-expressed genes of HOXA11

Co-expressed genes of HOXA11 in LUSC were collected from MEM (http://biit.cs.ut.ee/mem), cBioPortal (http://www.cbioportal.org), and GEPIA (http://gepia.cancer-pku.cn) for further evaluation. Genetic alterations of HOXA11, including amplification, deep deletion, and mRNA upregulation were additionally acquired from cBioPortal.

### Enrichment analysis and pathway annotation

Gathered genes were analyzed using bioinformatics. The enrichment of functions and signaling pathways of the target genes were analyzed using The Database for Annotation, Visualization and Integrated Discovery v6.8 (DAVID), FunRich, and Cytoscape. The String database (http://www.string-db.org) was applied to construct the protein-protein interaction (PPI) network for the hub gene identification. Moreover, hub genes were selected to obtain their expression and correlation with HOXA11 in LUSC. The immunohistochemistry results of the six hub genes in LUSC were retrieved from the Human Protein Atlas (HPA) database.

### Statistical analysis

Statistical analysis was conducted using SPSS 22.0. All data are shown as mean ± standard deviation (SD). An independent samples’ *t* test was adopted to examine the differences between cancer tissues and normal lung tissues. A one-way analysis of variance (ANOVA) was employed for analyzing differences of HOXA11 expression in various pathological gradings from in-house qRT-PCR data, as well as terms of M category, N category, race, and statuses of recurrence from the TCGA database. The relationships between the co-expressed genes and HOXA11 were assessed using the Pearson rank correlation, and the AUC was counted. To achieve an in-depth understanding of the prognostic value of HOXA11, we also used Kaplan-Meier curves to determine the survival time, including the OS and DFS. A two-sided *P* value < 0.05 was considered statistically significant.

## Results

### HOXA11 expression and clinicopathological features in LUSC using qRT-PCR

**Fig. 1:**
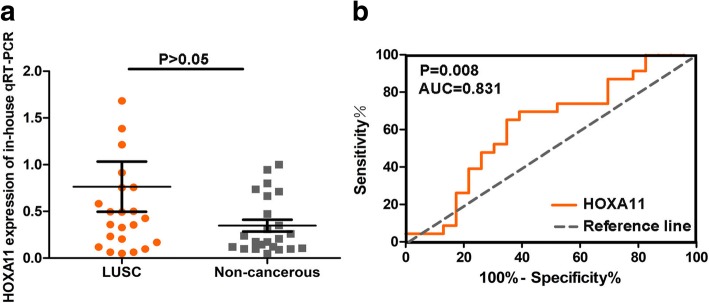
Data analysis of qRT-PCR. **a** The expression of HOXA11 in 23 LUSC and paired non-cancerous lung tissues. (**b**) The AUC of the TNM stage from the results of in-house qRT-PCR was 0.831 (*P* = 0.008)

The expression and clinicopathological features of HOXA11 in LUSC are displayed in Table 1. There was no significant correlation between HOXA11 expression and all clinical parameters. However, the expression of HOXA11 was upregulated in LUSC compared to in non-cancerous tissues, and the AUC of the TNM stage was 0.831 (*P* = 0.008) (Fig. 1a, b).

### Verification of HOXA11 expression in TCGA and Oncomine

**Fig. 2:**
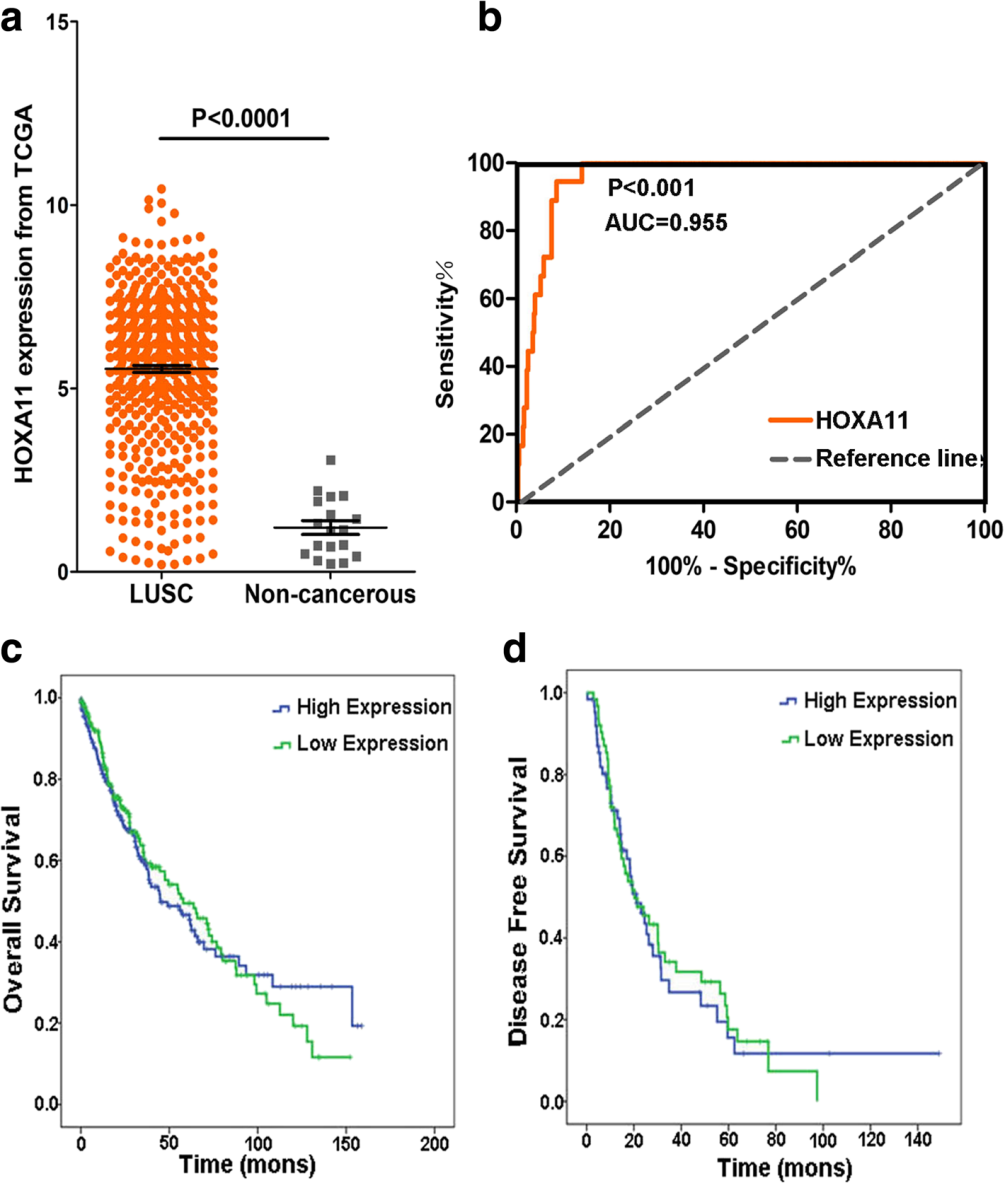
Data analysis of TCGA. **a** HOXA11 expressed higher in LUSC (5.531 ± 2.054) than that in non-cancer tissues (1.209 ± 0.813) from TCGA (*P* < 0.001). **b** The AUC of HOXA11 for diagnosing LUSC was 0.955 (*P* < 0.001). **c** The OS of LUSC patients (*P* = 0.795). **d** The DFS of LUSC patients (*P* = 0.864)

**Fig. 3:**
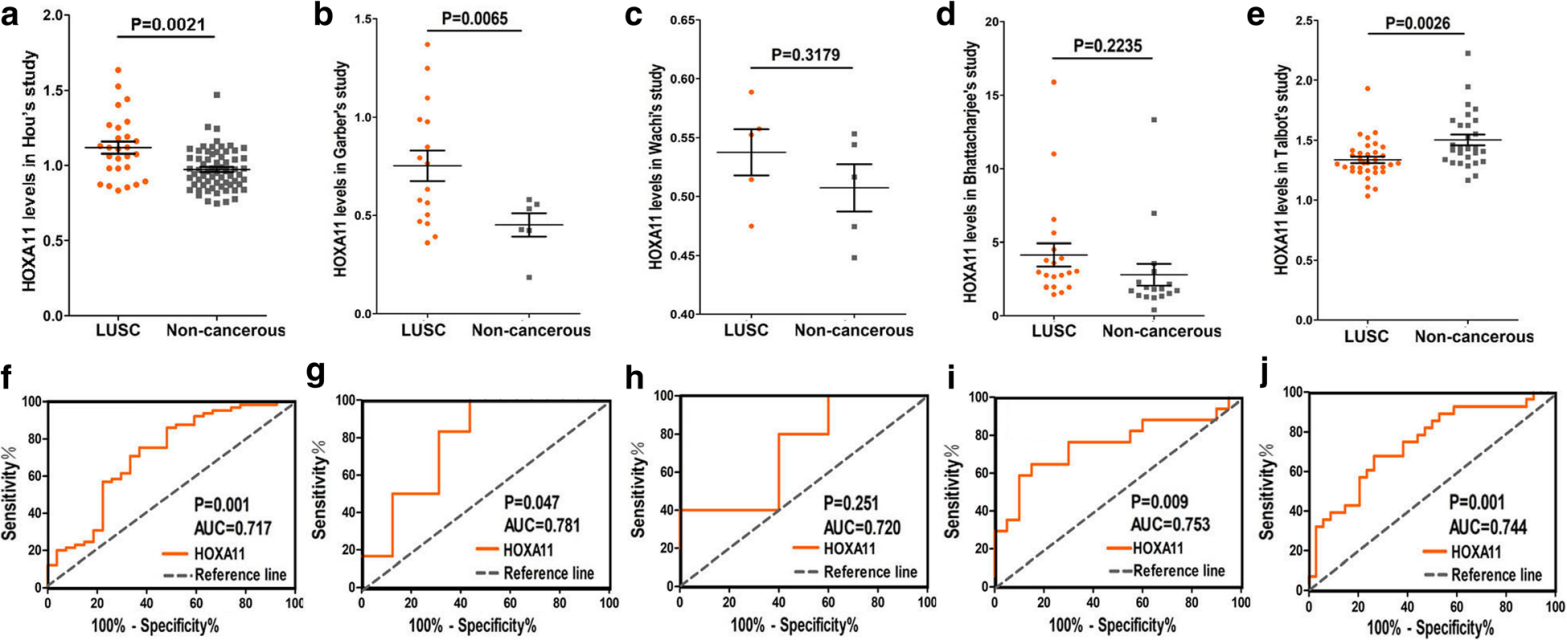
Data analysis of five studies extracted from Oncomine. **a** HOXA11 expressed higher in LUSC in Hou’s study (*P* = 0.002). **b** HOXA11 was higher in LUSC tissues in Garber’s study (*P* = 0.007). **c** HOXA11 expressed insignificant in Wachi’s study (*P* = 0.318). **d** HOXA11 showed no significant results in Bhattacharjee’s study (*P* = 0.224). **e** HOXA11 decreased in LUSC in Talbot’s study (*P* = 0.003). **f** The AUC of HOXA11 for diagnosing LUSC in Hou’s study was 0.717 (*P* = 0.001). **g** The AUC of HOXA11 for diagnosing LUSC in Garber’s study was 0.781 (*P* = 0.047). **h** The AUC of HOXA11 for diagnosing LUSC in Wachi’s study was 0.720 (*P* = 0.251). **i** The AUC of HOXA11 for diagnosing LUSC in Bhattacharjee’s study was 0.753 (*P* = 0.009). **j** The AUC of HOXA11 for diagnosing LUSC in Talbot’s study was 0.744 (*P* = 0.001)

**Fig. 4:**
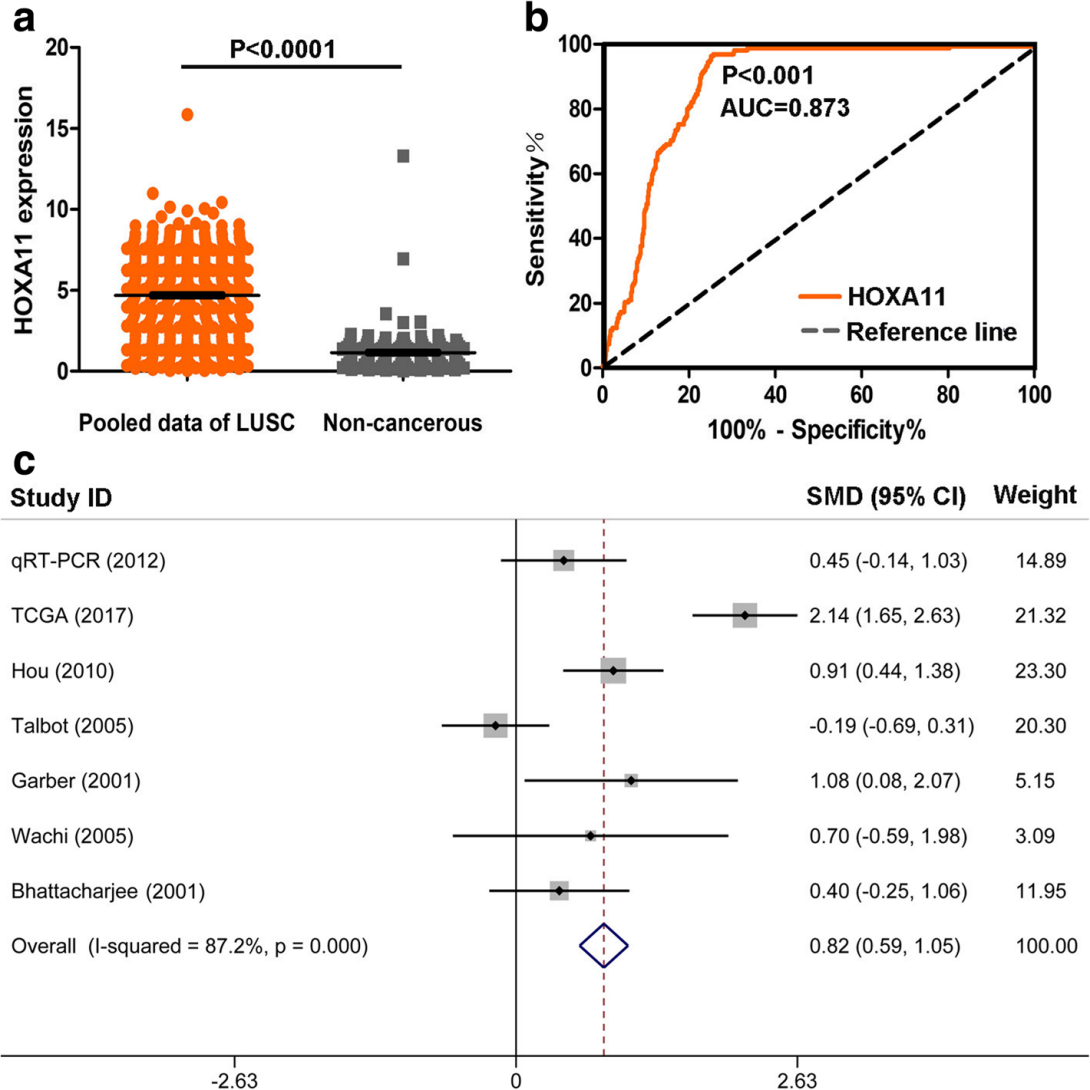
Analysis of pooled expressing profiles of HOXA11 in LUSC. **a** The expression of HOXA11 in 762 samples (600 LUSC and 162 non-cancerous lung tissues) from in-house qRT-PCR, TCGA, and Oncomine. **b** The AUC of HOXA11 for diagnosing LUSC was 0.873 (*P* < 0.0001). **c** The forest plots of HOXA11 levels in LUSC

As shown in Table 2, a cohort of 475 LUSC and 18 non-cancerous adjacent lung tissues was obtained from TCGA database. The results demonstrated that the expression of HOXA11 was noted higher in LUSC (5.531 ± 2.054) than in adjacent lung tissues (1.209 ± 0.813) (*P* < 0.001) (Fig. 2a). The AUC of the HOXA11 expression of LUSC for diagnosis was 0.955 (*P* < 0.001) (Fig. 2b). The expression of HOXA11 did not significantly differ in OS (*P* = 0.795) and DFS (*P* = 0.864) (Fig. 2c, d). As shown in Table 3, in the five studies of HOXA11 expression available at Oncomine, Hou et al. [25] and Garber et al. [26] found that HOXA11 expression in LUSC was significantly over-expressed (1.118 ± 0.211 vs 0.972 ± 0.135, *P* = 0.002; 0.752 ± 0.312 vs 0.451 ± 0.146, *P* = 0.006). The ROC curve analysis showed that the AUCs were 0.717 and 0.781 respectively, which were both significant (*P* < 0.05) (Fig. 3a, b, f, and g). Wachi et al. [27] and Bhattacharjee [28] illustrated that there were no noticeable differences between the HOXA11 expression in LUSC and adjacent normal tissues, but the expressions of HOXA11 was also upregulated (0.538 ± 0.044 vs 0.507 ± 0.045, *P* = 0.314; 4.141 ± 3.527 vs 2.796 ± 3.070, *P* = 0.228) (Fig. 3c, d). Meanwhile, the AUCs of Wachi’s and Bhattacharjee’s were estimated. There was no significant value of the AUC in the study of Wachi (AUC = 0.720, *P* = 0.251) (Fig. 3h). As for the study of Bhattacharjee, the AUC was 0.753 (*P* = 0.009) (Fig. 3i). In contrast, Talbot et al. [29] showed that HOXA11 was significantly downregulated (1.336 ± 0.161 vs 1.503 ± 0.236, *P* = 0.003), with an AUC of 0.744 (Fig. 3e, j). Additionally, as shown in Fig. 4a, scatterplots of pooled data demonstrated a remarkably higher level of HOXA11 in LUSC (4.700 ± 2.596 vs. 1.159 ± 1.217, *P* < 0.001). The AUC of pooled data was estimated to be 0.873 with *P* < 0.001 (Fig. 4b). The forest plots also revealed an increased HOXA11 in LUSC compared to non-cancerous lung tissues (SMD = 0.820, 95% CI = 0.594–1.046, *P* < 0.001) (Fig. 4c).

**Table 2:** Relationships between the HOXA11 level and clinicopathological parameters in LUSC based on the TCGA database

Clinicopathological parameters		*n*	Relevant expression of HOXA11 (2^-ΔCq^)		
			Mean ± SD	*t*	*P* value
Tissue	Adjacent non-cancerous lung tissue	18	1.209 ± 0.813	− 20.240^a^	1.5855E−17
	Cancer	475	5.531 ± 2.054		
Age (years)	< 60	41	5.578 ± 2.374	0.087^a^	0.931
	≥ 60	199	5.609 ± 2.080		
Gender	Male	353	5.593 ± 2.049	1.123^a^	0.262
	Female	120	5.349 ± 2.065		
Status	Dead	204	5.445 ± 2.003	− 0.799^a^	0.425
	Alive	269	5.597 ± 2.093		
Neoplasm cancer status	With tumor	108	5.335 ± 2.211	− 1.086^a^	0.278
	Tumor-free	299	5.590 ± 2.037		
M	M0	390	5.549 ± 2.069	*F* = 0.224^b^	0.800
	M1	4	6.041 ± 2.528		
	MX	73	5.431 ± 1.974		
T	T1–T2	385	5.526 ± 2.029	− 0.119^a^	0.906
	T3–T4	88	5.555 ± 2.172		
N	N0–N1	424	5.525 ± 2.050	*F* = 2.565^b^	0.078
	N2–N3	45	5.780 ± 1.974		
	NX	4	3.369 ± 2.596		
Race	White	331	5.465 ± 2.051	*F* = 3.892^b^	0.021*
	Asian	9	4.264 ± 2.662		
	Black	28	6.304 ± 1.585		
Recurrence	Distant metastasis	36	5.524 ± 2.346	*F* = 0.565^b^	0.571
	New primary tumor	12	5.457 ± 2.031		
	Locoregional recurrence	30	4.959 ± 2.135		

*P < 0.05 was considered statistically significant

^a^Student’s paired or unpaired *t* test was used for comparison between two groups

^b^One-way analysis of variance (ANOVA) was performed

**Table 3:** The expression of HOXA11 in five studies from the Oncomine database

	LUSC		Non-cancerous tissue		
Study	Mean ± SD	*n*	Mean ± SD	*n*	*P*
Hou	1.118 ± 0.211	27	0.972 ± 0.135	65	0.002
Garber	0.752 ± 0.312	16	0.451 ± 0.146	6	0.006
Wachi	0.538 ± 0.044	5	0.507 ± 0.045	5	0.314
Bhattacharjee	4.141 ± 3.527	20	2.796 ± 3.070	17	0.228
Talbot	1.336 ± 0.161	34	1.503 ± 0.236	28	0.003

### Achievement of co-expressed genes

**Fig. 5:**
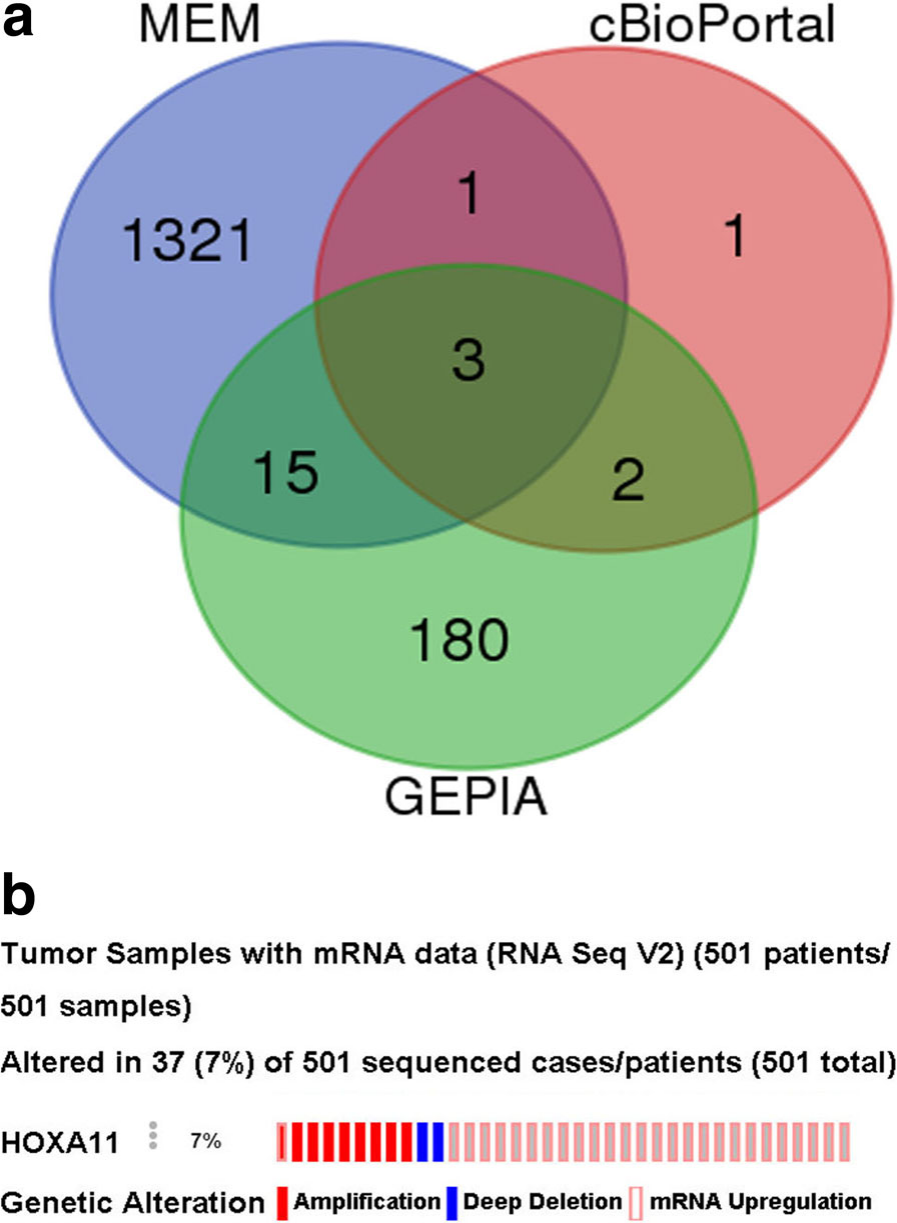
Diagrams of Venn and alterations of HOXA11 in LUSC. **a** The counts of intersected genes from MEM, cBioPortal, and GEPIA databases. **b** The alterative conditions of HOXA11 in LUSC obtained from the Oncomine database. Amplification and mRNA upregulation occurred on one patient at the same time

As shown in Venn, 1340 genes were obtained from MEM, 7 from cBioPortal, and 200 co-expression genes from GEPIA, respectively (Fig. 5a). Three genes, HOXA10, HOXA13, and HOXC10, were intersected in various platforms. Alterations of HOXA11 were indicated via cBioPortal, which showed that HOXA11 expression was upregulated in 27 LUSC patients, deleted in 2 LUSC patients, and amplified in 8 LUSC patients (Fig. 5b).

### Bioinformatics analysis

**Fig. 6:**
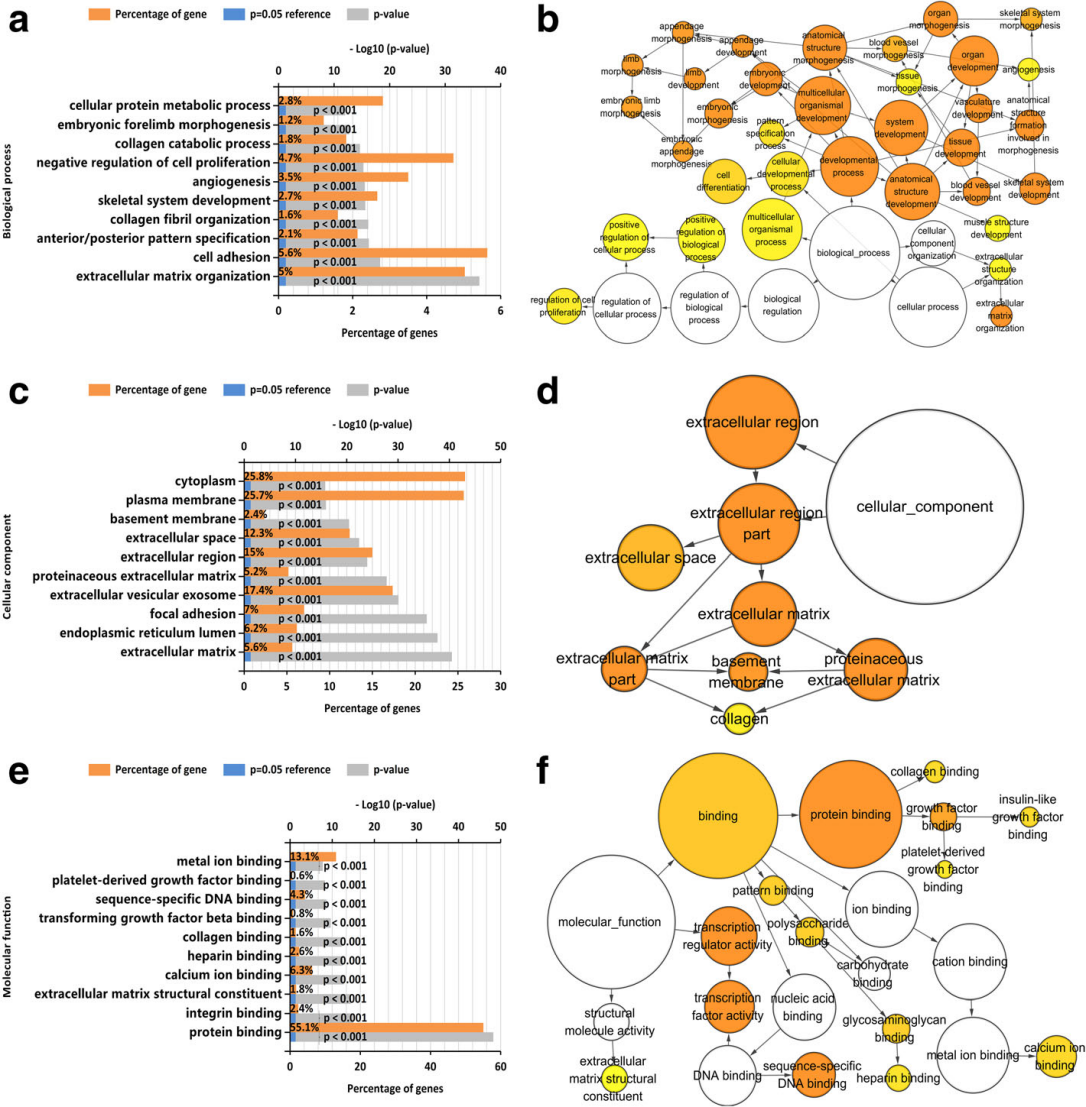
Top 10 significant pathways and GO enrichment analysis. **a** Graph of the 10 most significant pathways of BP category. **b** Enrichment analysis of BP, each node means one different function and more significant ones are filled in with a deeper color. **c** Top 10 significant terms in the CC category. **d** Enrichment analysis of the CC category; each node means one different function, and more significant ones are filled in with a deeper color. **e** Ten most valuable annotations of the MF category. **f** Enrichment analysis of the CC category; each node means one different function, and more significant ones are filled in with a deeper color

**Fig. 7:**
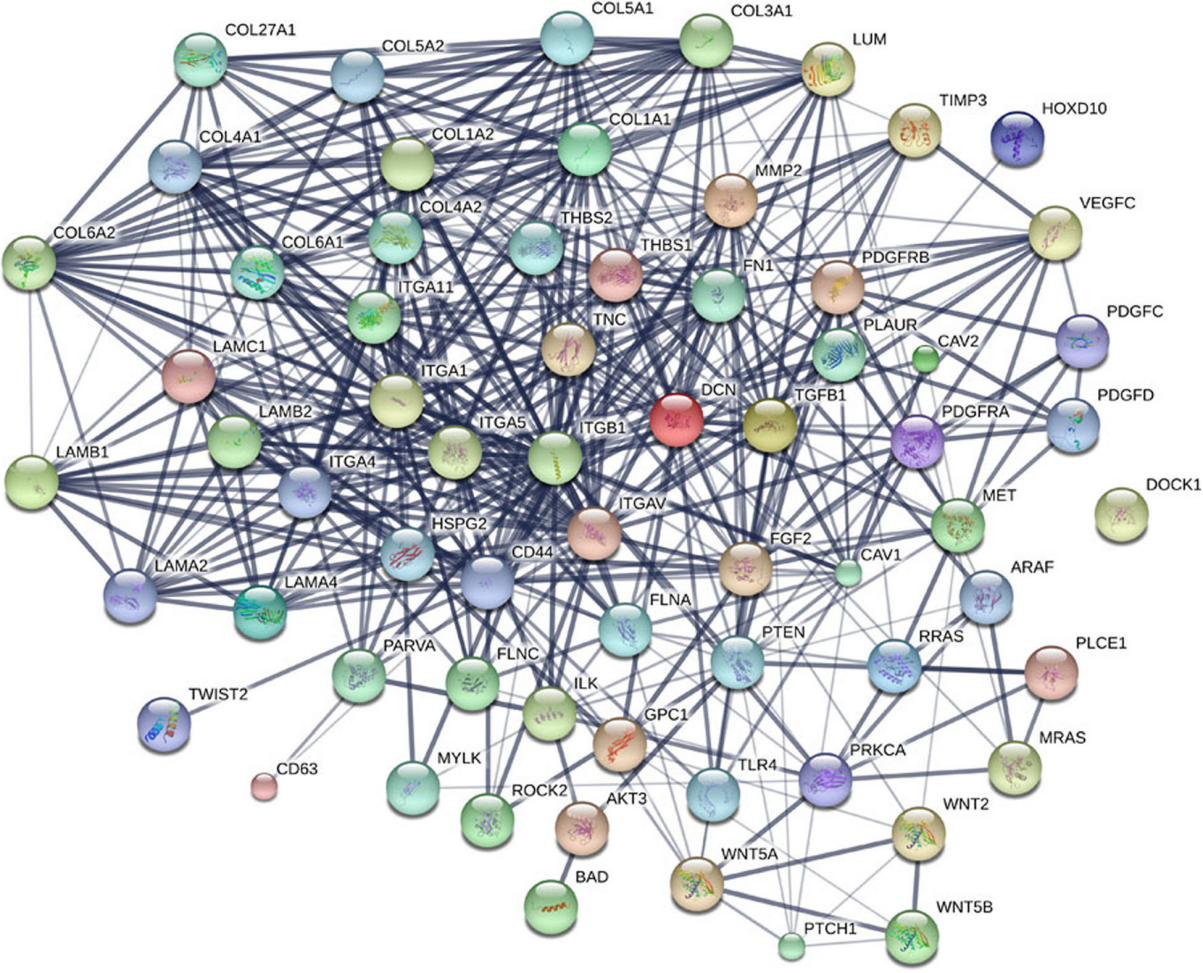
Interactions between different pairs of proteins. Nodes represent various symbols of genes; edges represent protein-protein associations

All analysis was based on the number of 1523 co-expression genes. The Gene Ontology enrichment analysis comprised three categories: a biological process (BP), a molecular function (MF), and a cellular component (CC). The most valuable 10 pathways of each category are presented in Fig. 6a, c, and 6e, including different kinds of functional relationship graphs (Fig. 6b, d, and f). For the KEGG pathways, the 10 most significant pathways are shown in Table 4. The PPI network is displayed (Fig. 7); three pairs of hub genes with the highest combined scores (PARVA, ILK, COL4A1, COL4A2, ITGB1, and ITGA5) were collected from the PPI network (Table 5).

**Table 4:** Top 10 significant KEGG pathway annotations

Term	Genes	Count	*P* value
Focal adhesion	CAV2, CAV1, TNC, COL3A1, ITGA11, PTEN, ITGB1, DOCK1, LAMB2, COL27A1, ITGAV, ILK, COL6A2, COL6A1, PDGFC, PDGFD, LAMB1, THBS1, THBS2, AKT3, FN1, PRKCA, COL4A2, COL4A1, ROCK2, MET, ITGA1, ITGA4, BAD, FLNC, COL5A2, COL5A1, FLNA, LAMA2, VEGFC, LAMA4, ITGA5, COL1A2, PDGFRA, PDGFRB, COL1A1, LAMC1, MYLK, PARVA	44	1.21E−12
ECM-receptor interaction	TNC, COL3A1, ITGA11, ITGB1, LAMB2, CD44, ITGAV, COL27A1, COL6A2, COL6A1, THBS1, LAMB1, THBS2, FN1, COL4A2, COL4A1, HSPG2, ITGA1, ITGA4, COL5A2, COL5A1, LAMA2, LAMA4, ITGA5, COL1A2, COL1A1, LAMC1	27	8.63E−12
Proteoglycans in cancer	WNT5A, CAV2, CAV1, WNT5B, MRAS, LUM, TLR4, DCN, MMP2, ITGB1, TIMP3, HOXD10, TGFB1, WNT2, CD44, ITGAV, RRAS, THBS1, FGF2, GPC1, TWIST2, AKT3, FN1, PRKCA, ROCK2, MET, HSPG2, CD63, FLNC, FLNA, PLAUR, PLCE1, ITGA5, ARAF, PTCH1	35	7.76E−08
Pathways in cancer	ADCY3, PPARD, PTGS2, GNA11, PPARG, MITF, LPAR1, MMP2, PTEN, GLI3, TGFB1, EDNRA, WNT2, CDKN2A, FGF2, AKT3, PRKCA, AR, PTGER3, ROCK2, RUNX1T1, VEGFC, PDGFRA, PDGFRB, LAMC1, WNT5A, WNT5B, GNAI1, KITLG, GNG11, GNG12, BDKRB2, ITGB1, LAMB2, ITGAV, RUNX1, LAMB1, TRAF5, FN1, BMP4, IL6, COL4A2, COL4A1, EPAS1, MET, TGFBR2, BAD, LAMA2, LAMA4, ADCY9, RASSF1, ARAF, PTCH1	53	1.75E−07
Amoebiasis	PRKCA, COL4A2, IL6, RAB7B, COL4A1, GNA11, COL3A1, TLR4, COL5A2, TGFB1, COL5A1, LAMA2, LAMA4, LAMB2, COL27A1, COL1A2, HSPB1, COL1A1, LAMC1, LAMB1, FN1	21	7.93E−06
PI3K-Akt signaling pathway	TNC, COL3A1, ITGA11, KITLG, TLR4, GNG11, GNG12, LPAR1, PTEN, ITGB1, LAMB2, COL27A1, ITGAV, COL6A2, CREB3L1, COL6A1, PDGFC, ANGPT1, PDGFD, LAMB1, THBS1, FGF2, THBS2, AKT3, FN1, PRKCA, IL6, COL4A2, COL4A1, MET, ITGA1, ITGA4, BAD, COL5A2, COL5A1, LAMA2, VEGFC, LAMA4, ITGA5, COL1A2, PDGFRA, PDGFRB, COL1A1, LAMC1	44	1.06E−05
Dilated cardiomyopathy	ADCY3, ITGA11, ITGA1, LMNA, CACNB3, ITGA4, TPM2, TPM1, ITGB1, TPM4, TGFB1, ADCY9, ITGA5, ITGAV, SGCD, CACNA1C, SGCB	17	5.69E−05
Hypertrophic cardiomyopathy (HCM)	IL6, ITGA1, LMNA, ITGA11, CACNB3, ITGA4, TPM2, TPM1, ITGB1, TPM4, TGFB1, ITGA5, ITGAV, SGCD, CACNA1C, SGCB	16	8.62E−05
Protein digestion and absorption	COL4A2, SLC8A1, COL4A1, COL3A1, COL15A1, COL5A2, COL5A1, COL7A1, COL27A1, PRSS3, COL6A2, COL1A2, COL12A1, COL6A1, COL1A1, DPP4	16	3.50E−04
Axon guidance	PLXNA3, NRP1, PLXNA1, ROCK2, GNAI1, MET, NTN4, EPHB3, ITGB1, SLIT2, SLIT3, SEMA5A, CFL2, PPP3CB, SEMA3C, NFATC4, SEMA3B, RHOD, SRGAP1	19	9.54E−04

**Table 5:** Top 10 pairs of hub genes from the PPI network

Node1	Node2	Homology	Co-expression	Experimentally determined interaction	Database annotated	Automated text mining	Combined score
PARVA	ILK	0	0.12	0.993	0.9	0.941	0.999
COL4A1	COL4A2	0.772	0.842	0.88	0.9	0.864	0.998
ITGB1	ITGA5	0	0.056	0.633	0.9	0.874	0.995
COL1A2	COL1A1	0.93	0.871	0.36	0.9	0.861	0.991
COL3A1	COL1A1	0.614	0.869	0	0.9	0.823	0.99
FN1	ITGAV	0	0	0.863	0.9	0.358	0.99
COL3A1	COL1A2	0.886	0.875	0	0.9	0.781	0.988
TIMP3	MMP2	0	0.082	0.528	0.8	0.871	0.987
COL1A2	LUM	0	0.724	0.36	0.9	0.323	0.986
TGFB1	DCN	0	0	0.387	0.9	0.797	0.986

### Expression and correlation of hub genes with HOXA11

**Fig. 8:**
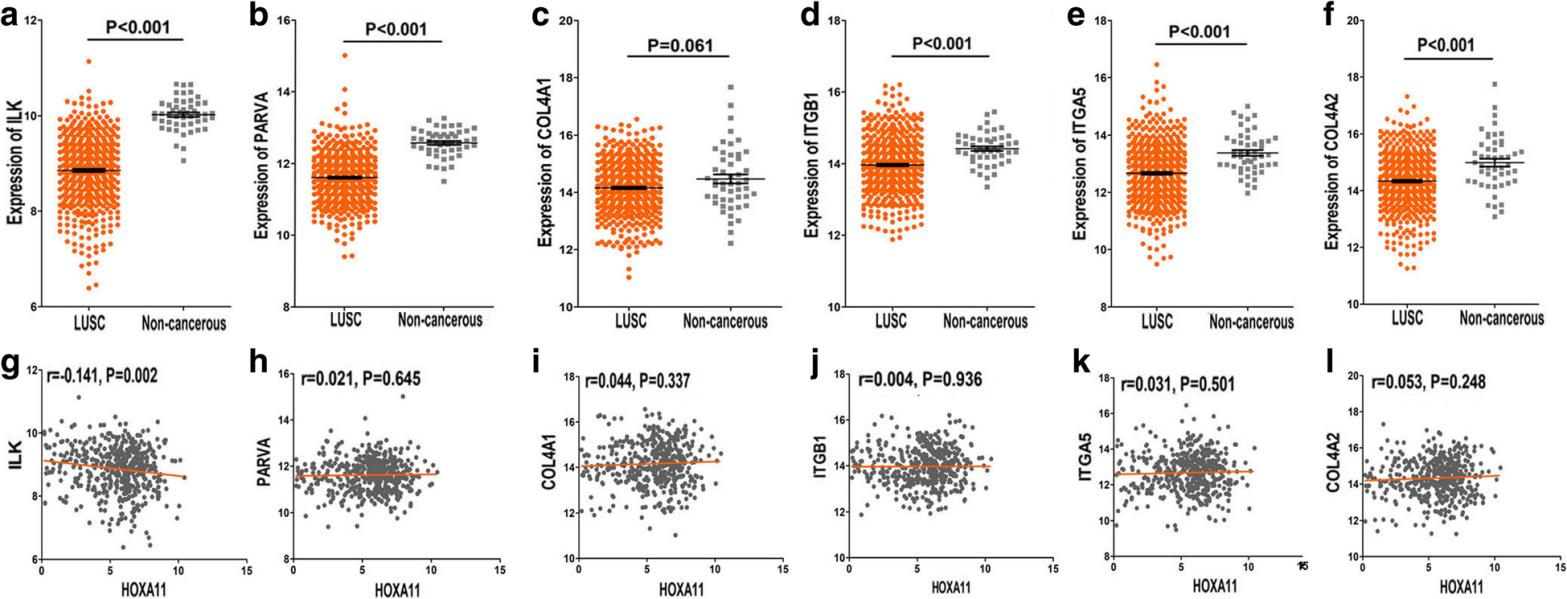
Hub genes’ expression in LUSC and correlations with HOXA11. **a** ILK was lower in LUSC tissues than in non-cancerous tissues (*P* < 0.001). **b** The gene PARVA was significantly overexpressed in normal tissues (*P* < 0.001). **c** The levels of COL4A1 in different tissues showed no significance (*P* = 0.061). **d** The hub gene ITGB1 revealed higher levels in normal tissues (*P* < 0.001). **e** ITGA5 was significantly decreased in LUSC tissues (*P* < 0.001). **f** COL4A2 upregulated in non-LUSC tissues (*P* < 0.001). **g** ILK and HOXA11 showed a negative correlation (*r* = − 0.141, *P* = 0.002). **h** Correlations between PARVA and HOXA11 showed no significance (*P* = 0.645). **i** Correlations between COL4A1 and HOXA11 showed no significance (*P* = 0.337). **j** Correlations between ITGB1 and HOXA11 showed no significance (*P* = 0.936). **k** Correlations between ITGA5 and HOXA11 showed no significance (*P* = 0.501). **l** Correlations between COL4A2 and HOXA11 showed no significance (*P* = 0.248)

**Fig. 9:**
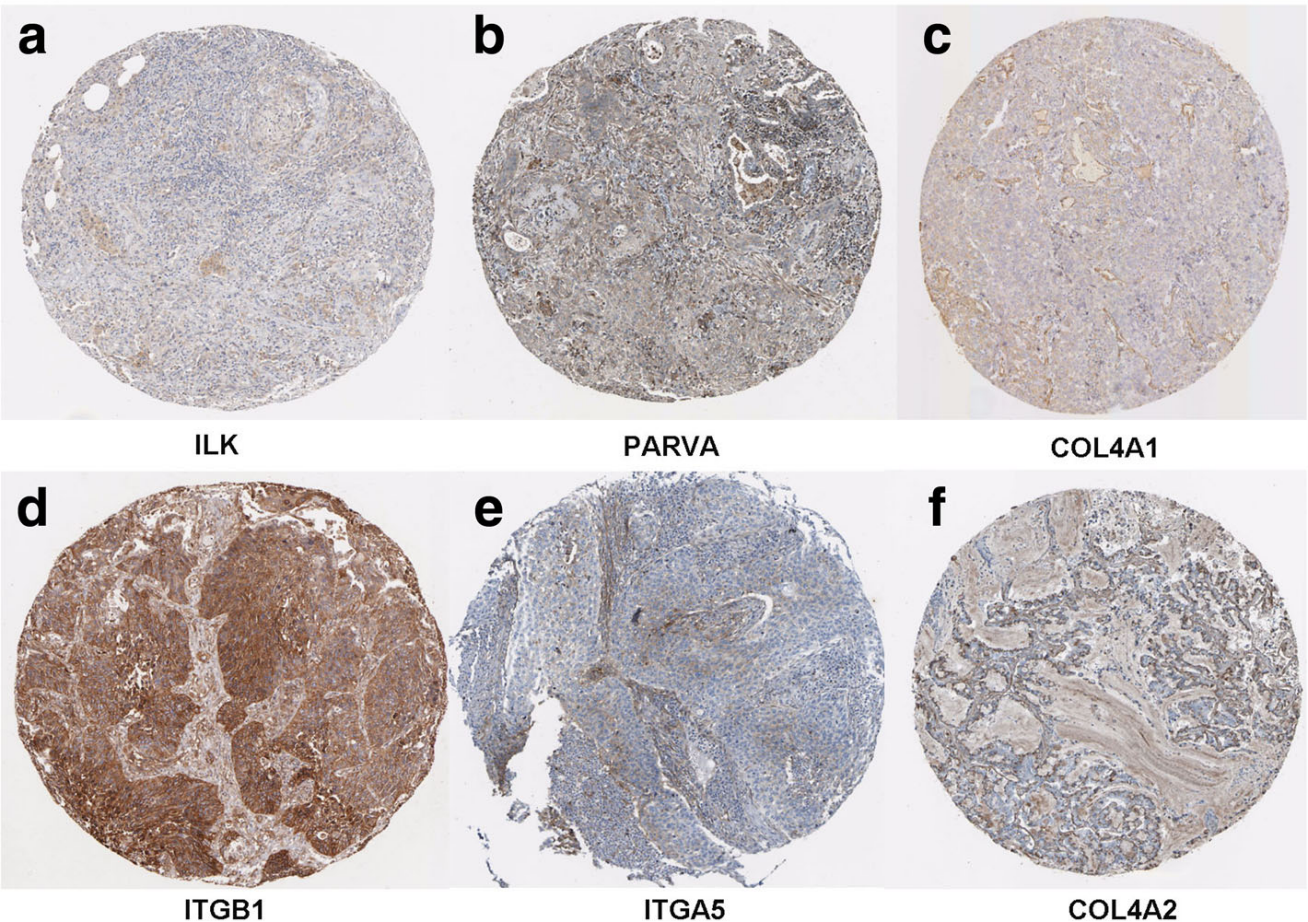
The protein level of the selected hub genes in LUSC tissues from The Human Protein Atlas. **a** ILK (Antibody CAB004041) expression in LUSC tissues. **b** PARVA (Antibody HPA005964) expression in LUSC tissues. **c** COL4A1 (Antibody CAB001695) expression in LUSC tissues. **d** ITGB1 (Antibody CAB003434) expression in LUSC tissues. **e** ITGA5 (Antibody CAB009008) expression in LUSC tissues. **f** COL4A2 (Antibody CAB010751) expression in LUSC tissues

Compared with non-cancerous lung tissues, the hub genes ILK, PARVA, COL4A2, ITGB1, and ITGA5 were significantly downregulated in LUSC (Table 6, Fig. 8a–f). Moreover, correlations between hub genes and HOXA11 were analyzed, and the gene ILK was negatively correlated with HOXA11 in LUSC (*r* = − 0.141, *P* = 0.002) (Fig. 8g–l). The HPA database indicated that there were lower levels of the six hub genes in NSCLC tissues: ILK (Antibody CAB004041), PARVA (Antibody HPA005964), COL4A1 (Antibody CAB001695), ITGB1 (Antibody CAB003434), ITGA5 (Antibody CAB009008), and COL4A2 (Antibody CAB010751) (Fig. 9a–f).

**Table 6:** Expressing profiles of six hub genes

	LUSC		Non-cancerous tissue		
	Mean ± SD	*n*	Mean ± SD	*n*	*P*
PARVA	11.61 ± 0.031	502	12.25 ± 0.054	49	< 0.001
ILK	8.852 ± 0.032	502	10.02 ± 0.048	49	< 0.001
COL4A1	14.15 ± 0.042	502	14.47 ± 0.159	49	0.061
COL4A2	14.33 ± 0.045	502	14.99 ± 0.138	49	< 0.001
ITGB1	13.97 ± 0.035	502	14.42 ± 0.063	49	< 0.001
ITGA5	12.67 ± 0.047	502	13.38 ± 0.103	49	< 0.001

## Discussion

HOX genes may play a central role in regulating gene expression, differentiation, and receptor signaling. Members of HOX family can also encode DNA-binding transcription factors [30, 31]. A growing body of studies observed that HOXA11 expression was downregulated in different tumor types. In glioblastoma, the decreased level of HOXA11 was confirmed as a significant marker of poorer prognosis. [32]. Moreover, Bai et al. suggested that the down-expressed HOXA11 gene may play an essential role in carcinogenesis by promoting gastric cancer development, which may be helpful to forecast the malignant behaviors of gastric cancer [33]. HOXA11 has been notably over-expressed in epithelial ovarian cancers [34]. Data from the current study, TCGA, Hou’s study [25], along with Garber’s study [26], all illustrated that the corresponding mRNA expression of HOXA11 is significantly over-represented in LUSC compared with adjacent tissues. The enhanced expression of HOXA11 might be a diagnostic target to use for distinguishing LUSC from healthy controls based on the ROC (AUC = 0.955, *P* < 0.001) from TCGA. Unfortunately, the expression detected by qRT-PCR revealed no significance. However, HOXA11 tends to be upregulated in LUSC compared to normal lung tissues. Interestingly, Talbot reported that the levels of HOXA11 were reduced in patients with LUSC. In light of these previous studies, HOXA11 expression seems to be higher in LUSC than in healthy controls. More LUSC specimens need to be collected to study the expression levels of HOXA11 in LUSC in the future. Among the co-expressed genes of HOXA11, a total of three genes, HOXA10, HOXA13, and HOXC10, were finally intersected, which all belong to the HOX gene family. HOXA10 is involved in gene expression, regulation, morphogenesis, and differentiation in ovarian carcinoma. HOXA10 and HOXA11 might be associated with primary tumors and specific histological subtypes [34]. HOXA13 regulates gene expression and differentiation. HOXA10, HOXA11, and HOXA13 might be useful targets to further mine the molecular pathogenesis of HOXA11 in early-stage lung adenocarcinoma [35]. Moreover, HOXC10 may act as the accelerator for original activation. In previous studies, HOXC10 has been reported to be associated with the increased invasion of malignancies [36, 37]. These three co-expressed genes and HOXA11 might play several pivotal roles in LUSC, such as the subtype differentiation of lung cancer, the regulation of LUSC progression, and the development of efficient therapeutic strategies.

The pathway of focal adhesion has been extensively reported to be the most significant pathway in various diseases and signaling pathways as well. It regulates diverse cellular functions that were once activated by focal adhesion kinase (FAK), including adhesion, proliferation, migration, and survival [38–40]. A pattern of enhanced expression of FAK in lung carcinomas has been reported, which is related to nodal involvement and the deterioration of advanced disease stages [41, 42]. Therefore, FAK protein expression may help in predicting the aggressive behavior of LUSC. Meanwhile, FAK might be pursued as a promising therapeutic target for LUSC.

Six hub genes were analyzed, PARVA, ILK, COL4A1, COL4A2, ITGB1, and TIGA5. Except COL4A1 gene, the rest of the hub genes were all significantly decreased in LUSC compared to adjacent healthy controls. COL4A1 and COL4A2 both belong to the type IV collagen gene family. Both can act as inhibitors of angiogenesis and tumor growth. In LUSC, they can control vascular invasion and inhibit the size of tumors. There is more reason to believe that the upregulated HOXA11 gene plays a carcinogenic role in LUSC. HOXA11 might depress COL4A1 and COL4A2 expression levels in LUSC. ITGB1 has been recognized in the processing of metastatic diffusion of tumor cells, and ITGA5 may promote tumor invasion. In lung cancer, higher expression of ITGA5 may be correlated with a shorter survival time. Meanwhile, previous studies have indicated that modulating the ILK signaling pathway by PARVA made it more vulnerable to metastasis in lung adenocarcinoma [43]. These hub genes might also similarly interact with each other via various signaling pathways in LUSC. Thus, we speculate that LUSC patients’ prognosis might be better and their survival time might be longer than for patients with other subtypes of lung cancer. The mechanisms and functions between hub genes and HOXA11 in LUSC remain elusive and need to be validated in the future.

## Conclusion

The findings of the present study indicate that HOXA11 high expression might lead to the occurrence and development of LUSC. Meanwhile, co-expressed HOXA11 genes may influence the prognosis of LUSC, and the gene ILK may have the complete reverse functions in LUSC compared with HOXA11. Hub genes need to be further analyzed to ensure their mechanisms and functions in LUSC.

## Abbreviations

ANOVA: Analysis of variance
BP: Biological process
CC: Cellular component
COL4A1: Collagen type IV alpha 1 chain
COL4A2: Collagen type IV alpha 2 chain
DFS: Disease-free survival
FAK: Focal adhesion kinase
GO: Gene Oncology
HOXA11: Homeobox A11
ILK: Integrin-linked kinase
ITGA5: Integrin subunit alpha 5
ITGB1: Integrin subunit beta 1
KEGG: Kyoto Encyclopedia of Genes and Genomes
LUAD: Lung adenocarcinoma
LUSC: Lung squamous cancer
MF: Molecular function
NSCLC: Non-small cell lung cancer
OS: Overall survival
PARVA: Parvin alpha
PPI: Protein-protein interaction
ROC curve: Receiver operating characteristic curve
SD: Standard deviation
TCGA: The Cancer Genome Atlas
